# Assessment of phylogenetic informativeness in mitochondrial and nuclear genes for mammalian systematics using sparse learning

**DOI:** 10.3389/fbinf.2025.1704212

**Published:** 2026-01-08

**Authors:** Carlos G. Schrago, Beatriz Mello

**Affiliations:** Department of Genetics, Federal University of Rio de Janeiro, Rio de Janeiro, Brazil

**Keywords:** phylogenetic informativeness, phylogenetic signal, mitochondrial genome, species delimitation, molecular taxonomy, sparse learning, Lasso regression

## Abstract

Despite the growing availability of nuclear genomic data, mitochondrial genes remain the most widely used molecular markers in mammalian systematics. However, a quantitative assessment of the phylogenetic information content of mitochondrial loci compared to nuclear loci has never been carried out. Here, we apply a sparse learning approach based on Lasso regression to evaluate the contribution of alignment sites to phylogenetic likelihoods, providing the first estimates of phylogenetically effective lengths for markers commonly used in mammalian systematics. Analyzing more than 30,000 complete mammalian mitochondrial genomes and nuclear panels composed of either 100 randomly selected complete coding sequences or of partial gene segments from conventional markers, we examined phylogenetic informativeness at two taxonomic levels: within-species and among-species. On average, ∼32% of mitochondrial sites and ∼38% of nuclear sites were classified as phylogenetically informative. We found that the number of phylogenetically informative sites were positively correlated with total gene length. Therefore, longer mitochondrial genes, particularly *ND5*, *COX1*, and *CYTB*, harbored the largest numbers of informative sites. Although nuclear coding sequences contained, on average, more informative sites, mitochondrial genes also yielded consistent resolution of among-species relationships. Overall, our results provide the first large-scale, quantitative comparison of phylogenetic information content across mammalian mitochondrial and nuclear genes, offering a principled framework for marker selection in future systematics studies that can be broadly applied to any lineage.

## Introduction

In mammalian systematics, as in many other animal lineages, mitochondrial genes remain widely used to investigate evolutionary history despite the increasing affordability of whole-genome sequencing (Mackiewicz et al., 2019; Zhang et al., 2021; 2023). Their use is prevalent in shallow-level taxonomic studies, such as those involving populations and closely related species (Phillips et al., 2022; Carrión-Olmedo and Brito, 2025; Ribeiro et al., 2025). Among these, the mitochondrial genes cytochrome *b* (*CYTB*) and the cytochrome oxidase I (*COX1*) have been partially or fully sequenced for more than 5,000 and 3,000 mammalian species, respectively. For both markers, more than 80% of the species have multiple individuals sequenced, allowing within-species analyses (NCBI, https://www.ncbi.nlm.nih.gov, as of September 2025). In contrast, for mammalian nuclear genes, only humans have been sampled at a comparable scale. The number of articles employing mitochondrial genes that address mammalian taxonomic problems has grown exponentially over the years (Scopus, https://www.scopus.com, as of September 2025). Therefore, until genome sequencing becomes substantially more affordable and current bioinformatics bottlenecks are mitigated, mitochondrial genes are likely to remain a key resource for systematics.

Surprisingly, although mitochondrial genes are extensively used in mammalian systematics, few studies have specifically assessed their information content for resolving evolutionary relationships in mammals (Zardoya and Meyer, 1996; Corneli and Ward, 2000, p. 200; Duchêne et al., 2011). Therefore, the reliance on mitochondrial loci in mammalian systematics has been guided largely by tradition. Indeed, many studies have adopted operational criteria, such as genetic distance thresholds, for species delimitation, which are seldom critically evaluated (Bradley and Baker, 2001; Schrago and Mello, 2020). To date, no large-scale analysis has formally compared the efficacy of mammalian mitochondrial genes with that of nuclear genomic regions.

This gap largely reflects the absence of methodological approaches capable of addressing systematists’ central questions. For instance, given a set of genes, which one carries the most phylogenetic information for both topology and branch length estimation? Despite their importance, such operational questions have received relatively little attention from statistical biologists. Previous efforts have mainly focused on contrasting the fit of competing topologies (Kishino and Hasegawa, 1989; Shimoidara, 2002). Some studies have introduced metrics like the phylogenetic informativeness profiles (Townsend, 2007) or have employed log-likelihood comparisons across candidate trees (López-Giráldez et al., 2013). However, these approaches typically rely on predefined hypotheses or reference topologies, limiting their suitability for general, data-driven assessments of phylogenetic information content.

Recently, Kumar and Sharma (Kumar and Sharma, 2021) have demonstrated the utility and flexibility of sparse learning methods in phylogenetics, such as the Lasso regression (Tibshirani, 1996). The Lasso regression represents a supervised machine learning approach that performs automatic feature selection by penalizing less informative predictors. Following the treatment presented by Ecker et al. (Ecker et al., 2022), we proposed a method to estimate the number of phylogenetically informative alignment sites using a supervised sparse learning approach based on the Lasso (Schrago, 2025). This method weights the contribution of likelihoods of individual alignment sites to the overall tree likelihood by generating an ensemble of random tree topologies and calculating site-wise log-likelihoods under a probabilistic model. Sites that consistently impact the phylogenetic likelihood, including topology and branch lengths, are identified and considered as phylogenetically informative. This approach provides a principled means of quantifying phylogenetic informativeness and defining phylogenetically effective gene length that is probabilistic and topology-agnostic, thereby enabling direct comparisons among loci and facilitating filtering schemes to improve phylogenetic inference.

In this work, we used this Lasso approach to conduct a large-scale analysis of phylogenetic informativeness across more than 30,000 mammalian mitochondrial genomes and two sets of nuclear loci, one consisting of a random sample of coding sequences from complete genomes, and another comprised of partial genes conventionally used in mammalian systematics. Together, these datasets allow us to provide the first comprehensive, comparative assessment of the phylogenetic information content of these genomic regions using statistical learning.

## Materials and methods

### Assembly of datasets and phylogenetic analysis

To assess the information content of mitochondrial and nuclear genes in mammals, we downloaded a total of 30,603 complete mammalian mitochondrial genomes from the NCBI database. To prevent over-representation of human sequences, all species of the genus *Homo* were excluded. Besides the widely used *CYTB* and *COX1* genes, we analyzed all mitochondrial protein-coding genes longer than 700 bp in humans (*CYTB*, *COX1*, *COX2*, COX3, *ND1*, *ND2*, *ND4*, and *ND5*) to avoid including very short sequences that are less commonly used in phylogenetic studies. We also analyzed a sub-dataset combining the *CYTB* and *COX1* genes, as these are the most frequently sequenced mitochondrial markers in mammalian systematics.

Using NCBI-informed taxonomy ranks, we focused on the two taxonomic levels at which mitochondrial genes are commonly employed in mammalian systematics: (*i*) within-species, corresponding to populational divergences, and (*ii*) among-species, encompassing divergences among species within the same genus. These levels are hereafter referred to as within-species and among-species, respectively. At the within-species level, we gathered data for 122 species, each represented by at least 05 mitochondrial genomes. At the among-species level, we investigated 100 genera, each with mitochondrial genomes available for at least 05 species. In this case, if multiple mitochondrial genomes were available for a given species, one genome was randomly selected. Consequently, a total of 976 (122 species × 8 genes), and 800 (100 genera × 8 genes) alignments were assembled.

For comparison with nuclear genes, we downloaded 17,439 ortholog alignments of coding sequences (CDS) for more than 500 mammalian species assembled employing the TOGA (Tool to infer Orthologs from Genome Alignments) pipeline (Kirilenko et al., 2023). To ensure comparability between datasets, we selected nuclear genes with coding sequence lengths ranging from 700 to 2,000 bp. Because the availability of complete mammalian nuclear genomes for within-species analyses is limited, the nuclear-mitochondrial comparison was restricted to the among-species level. Within the TOGA data set, we identified eight genera represented by more than five species with sequenced genomes: *Balaenoptera* (*physalus*, *bonaerensis*, *edeni*, *musculus*, *acutorostrata*), *Bos* (*gaurus*, *taurus*, *mutus*, *indicus*, *grunniens*, *frontalis*), *Marmota* (*marmota*, *monax*, *flaviventris*, *himalayana*, *vancouverensis*), *Mus* (*caroli*, *musculus*, *spretus*, *pahari*, *spicilegus*), *Myotis* (*septentrionalis*, *lucifugus*, *davidii*, *myotis*, *brandtii*), *Ovis* (*nivicola*, *orientalis*, *aries*, *canadensis*, *ammon*), *Panthera* (*leo*, *pardus*, *uncia*, *onca*, *tigris*), and *Peromyscus* (*californicus*, *crinitus*, *polionotus*, *maniculatus*, *leucopus*, *eremicus*). A total of 2,466 nuclear genes within the target length range were common to all species listed above. Due to computational constraints, we analyzed a random subset of 100 genes from this pool, resulting in 800 alignments (8 genera × 100 genes).

Finally, to compare our results with common practice in mammalian taxonomy, we surveyed all mammalian sequences deposited in the NCBI database to identify which nuclear loci are most frequently sequenced. Segments of the *IRBP*, *BRCA1*, *RAG1*, and *vWF* genes were the most widely represented. We therefore assembled an independent panel composed of these conventional nuclear markers, including alignments that captured both within-species and among-species levels of diversity (details in Table 1).

**TABLE 1:** Number of alignments and average alignment length of commonly used nuclear genes.

Gene	Taxonomic level	Number of alignments examined	Average alignment length (95% quantile)^a^
IRBP	Within species	224	1,173.7 (757.7–1,647.4)
	Among species	74	1,266.4 (861.4–1,698.0)
BRCA1	Within species	105	1,025.6 (737.4–1829.0)
	Among species	28	1,356.1 (785.6–2,560.8)
RAG1	Within species	160	1,173.3 (760.6–2085.7)
	Among species	58	1,498.4 (773.1–2,925.9)
vWF	Within species	6	1,028.2 (849.4–1,198.4)
	Among species	17	1,120.3 (916.2–1,302.6)

^a^ Alignments lengths including gaps.

In total, we analyzed four datasets: two were composed of genomic samples – a set of mitochondrial protein-coding genes and another of nuclear CDS obtained through TOGA; and two reflecting commonly used markers in mammalian systematics – a combined *CYTB* + *COX1* mitochondrial dataset and the panel of conventional nuclear loci (*IRBP*, *BRCA1*, *RAG1*, and *vWF*), which are hereafter referred to as the conventional mitochondrial and conventional nuclear marker datasets, respectively.

To facilitate site-level mapping, we first generated, for each gene investigated, a single alignment that combined sequences from both the within-species and among-species samples. Sequences were then separated back into their respective within-species and among-species groupings for all downstream analyses. Sequence alignments for mitochondrial genes and partial nuclear genes were performed with MAFFT 7.4 (Katoh and Standley, 2013) using translated amino acid sequences when feasible to preserve reading frames. All maximum likelihood (ML) phylogenetic inference was conducted with IQ-TREE2 (Minh et al., 2020), employing the substitution model selected by the ModelFinder algorithm (Kalyaanamoorthy et al., 2017). ML topologies were estimated to compute branch supports, which were assessed by the parametric approximate likelihood-ratio test (aLRT) statistic (Anisimova and Gascuel, 2006). In total, 2,576 IQ-TREE analyses were performed.

### Inference of phylogenetically informative sites

Lasso regression is a supervised machine learning approach that can be used to identify phylogenetically informative sites by modeling how the likelihood of each alignment column (sites) contributes to the overall likelihood of a phylogenetic tree (Schrago, 2025). Hence, the method highlights only the most influential predictors (alignment sites) of the tree topology. To do so, for a given sequence alignment, thousands of random phylogenetic trees are first generated, and the log-likelihood of each site is calculated under each tree (topology and branch lengths). These site-wise log-likelihoods are then used as explanatory variables (features) in a regression model, with the total log-likelihood of each tree as the response variable. Because of the penalty imposed by Lasso on regression coefficients ( β ), many sites receive coefficients of exactly zero, indicating no meaningful contribution to the tree likelihood. Sites with non-zero coefficients are classified as phylogenetically informative. This framework enables an objective identification of informative sites without relying on predefined topologies or arbitrary filtering thresholds.

Formally, we define the phylogenetically effective gene length of a gene *g* with *n* alignment sites (PEGL) as the total number of alignment sites assigned non-zero coefficients ( $\hat{\beta }\neq 0$ ) in the Lasso regression model under the penalization $\lambda^{*}$ selected by cross-validation:

$$PEGL\left(g\right)=\sum_{i=1}^{n}1\left\{\hat{\beta_{i}}\left(\lambda^{*}\right)\neq 0\right\}$$

This quantity represents the subset of sites that meaningfully contribute to the overall phylogenetic likelihood, providing a direct and probabilistic estimate of gene’s effective length, independent of any assumed topology or arbitrary filtering thresholds. To facilitate comparisons among loci of different sizes, we report the frequencies of informative sites, i.e., PEGL(*g*)/*n*.

Lasso analysis was carried out independently for each gene alignment using the *glmnet* package of R (Friedman et al., 2010). For each alignment, we generated 10,000 random trees (including both topology and branch lengths) using IQ-TREE2 (Minh et al., 2020), employing the “-r” flag and the “--sitelh” command to record site-wise log-likelihoods. This set of random trees provided the observations used to estimate the β coefficients of the linear model.

We compared the information content of coding sequences using three metrics: (i) the proportion of informative sites (PEGL(*g*)/*n*), (ii) the number of sites identified by Lasso as phylogenetically informative (the effective gene length), and (iii) the average parametric aLRT across internal branches of ML phylogenies. The aLRT statistic was included because it depends solely on tree topology, whereas the Lasso-based measure reflects contributions to both topology and branch lengths. Additionally, for the widely used *CYTB* and *COX1* genes, we mapped the distribution of informative sites along each coding sequence to highlight regions enriched in phylogenetic signal.

Lastly, to test whether the mean percentage of informative sites differed between mitochondrial and nuclear genes, we performed a non-parametric bootstrap analysis at the among-species level. Values from all mitochondrial genes were pooled into a single vector, and values from nuclear genes were treated as a second vector. We then generated 10,000 bootstrap replicates of the mean difference between the two groups using the *boot* package (Canty and Ripley, 2025) in R. The 95% confidence interval of the difference was obtained using the percentile method (*boot.ci*). A difference was considered statistically significant if the confidence interval did not include zero. This approach is robust to deviations from normality and enables inference on mean differences without relying on parametric tests.

## Results

**FIGURE 1:**
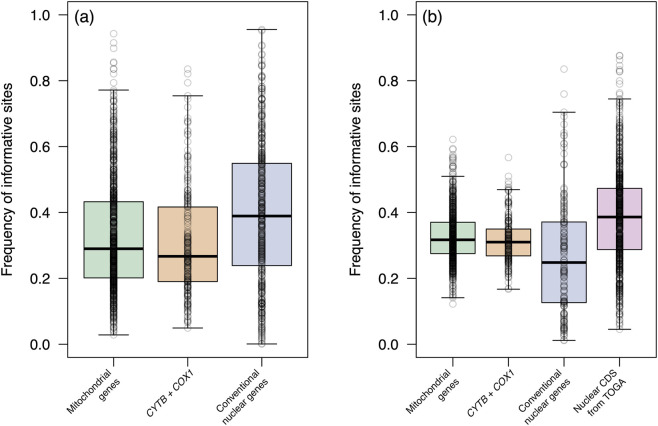
Distribution of the frequency of phylogenetically informative sites across each data set studied. **(a)** Within-species and **(b)** among-species alignments. Each point represents an alignment, showing the proportion of sites inferred as informative by Lasso regression.

In mammalian mitochondrial genes, regardless of the taxonomic level, sparse learning with the Lasso method identified on average 32.7% of alignment sites as informative to tree likelihood. For *COX1* and *CYTB*, this frequency averaged 31.1% (Figures 1a,b). At the within-species level, the proportion of informative sites ranged from 2.8% to 94.3% (mean = 32.7%) when all mitochondrial genes were considered, whereas the mean dropped to 30.6% for *CYTB* and *COX1* (8.3%–69.0%). In contrast, conventional nuclear genes displayed higher frequency of informative sites at this level, with the mean proportion of informative sites at 39.6% (4.2%–81.3%) (Figure 1a).

At the among-species level, the frequency of sites informative to the tree likelihood of mitochondrial genes ranged from 12.2% to 62.2% (mean = 32.8%), while the combined *CYTB* + *COX1* dataset ranged from 20.6% to 47.4% (mean = 31.8%). Nuclear genes randomly sampled from TOGA alignments, which were restricted to the among-species level analysis, exhibited, on average, a higher mean frequency (38.7%), with values ranging from 4.5% to 87.7%. Conventional nuclear genes, on the other hand, performed more poorly at this level, with a frequency of 26.5% informative sites (3.3%–65.7%) (Figure 1b).

The variance in the frequency of informative sites differed across datasets. At the among-species level, mitochondrial genes showed relatively lower variance (sd = 7.9%) compared with both nuclear datasets (14.7% for TOGA CDS and 17.2% for conventional nuclear markers). A similar pattern was observed at the within-species level, where nuclear genes showed higher variance (sd = 21.2%) than mitochondrial genes (16.6%). Bootstrap analysis indicated that the mean percentage of informative sites did not differ significantly (*p* = 0.501) between the two genomic panels (mitochondrial genes and nuclear CDS from TOGA). The 95% percentile confidence interval of the difference included zero, confirming no detectable difference between groups (Supplementary Figure S1).

**FIGURE 2:**
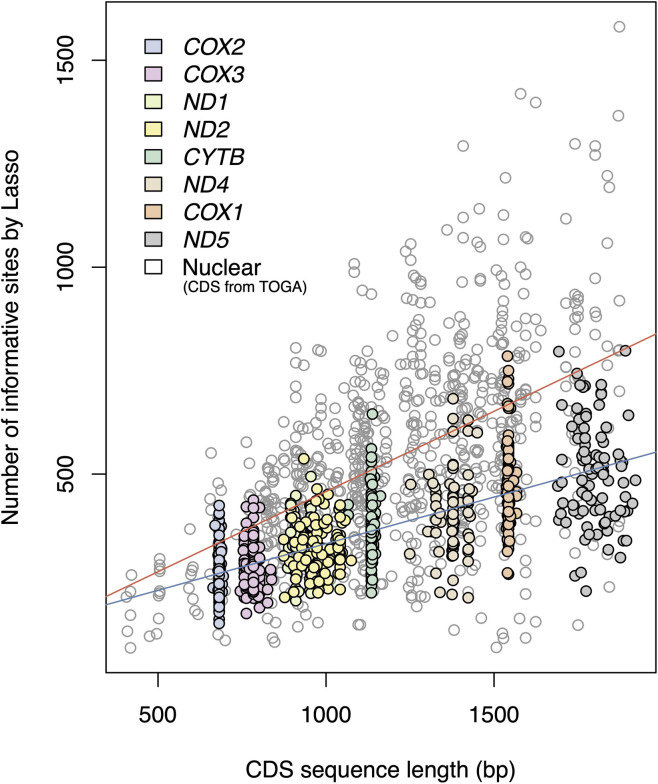
Gapless coding sequence length (bp) versus number of informative sites inferred by Lasso for among-species alignments. Regression lines are shown for mitochondrial genes (blue, *R* ^2^ = 0.48, *p* < 0.01) and nuclear genes (red, *R* ^2^ = 0.27, *p* < 0.01).

Across genomic compartments, longer genes generally contained more sites classified as informative (Figure 2). The number of sites classified as informative by Lasso was positively correlated with gapless sequence length in both mitochondrial and nuclear genes (*p* < 0.01). The correlation was stronger for mitochondrial genes (*r* = 0.70) than for nuclear genes (*r* = 0.52). However, the rate of information gain per additional site was lower in mitochondrial genes, with slopes of 0.23 and 0.36 for mitochondrial and nuclear genes, respectively. Consequently, for a given sequence length, nuclear genes tended to contain a greater number of phylogenetically informative sites.

**FIGURE 3:**
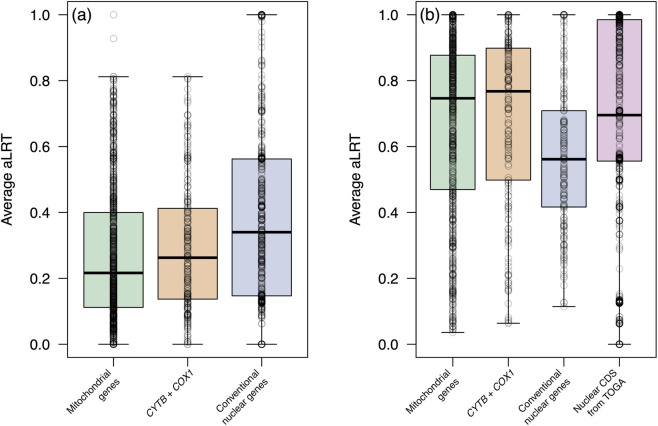
Average aLRT values for within-species **(a)** and among-species **(b)** datasets. Each point represents an alignment, showing the mean of the aLRT values estimated in IQ-TREE for that alignment.

For within-species datasets, average aLRT values rarely exceeded 0.8. In contrast, for among-species datasets, average aLRT values for above 0.75 were frequent, except for conventional nuclear markers. At this level, both mitochondrial sets exhibited average aLRT values (∼0.67) comparable to nuclear genes from TOGA (0.67), whereas the panel with conventional nuclear genes had an average aLRT of 0.57. Variance of average aLRT values was highest for TOGA CDS (sd = 0.31) and lowest for conventional nuclear genes (sd = 0.22), with both mitochondrial datasets showing similar intermediate values (sd = 0.26). For resolving relationships among congeneric species,30.5% of TOGA CDS alignments achieved an average aLRT ≥ 0.95, compared with 10.3% for mitochondrial genes, 11.3% of *CYTB* + *COX1*, and 7.3% for conventional nuclear genes (Figure 3).

**FIGURE 4:**
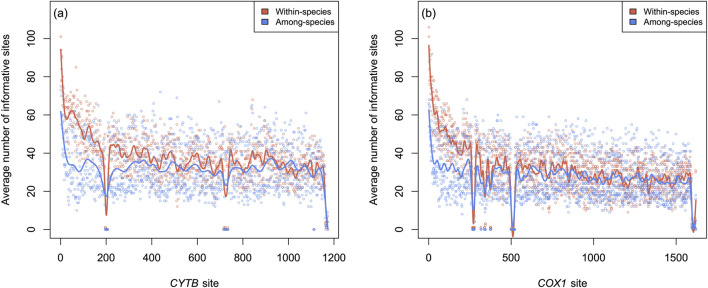
Distributions of Lasso-identified informative sites along **(a)** *CYTB* and **(b)** *COX1* coding sequences for both levels of genetic diversity investigated: within-species and among-species of the same genera.

For both *COX1* and *CYTB*, the distribution of phylogenetically informative sites varied slightly depending on the taxonomic level investigated (Figure 4). When resolving evolutionary relationships within species, the first halves of both genes were generally more informative. In contrast, for resolving among-species phylogenies, phylogenetic information was more evenly distributed across the coding sequence. Notably, some regions appeared to provide very little information for resolving both within-species and among-species relationships (low valleys in Figure 4). This artifact resulted from indel-rich sites, which arose because alignments were performed at the genus level rather than separately for individual species, in order to facilitate the mapping of informative sites across datasets.

## Discussion

We showed that, on a genome-wide scale, nuclear genes exhibit greater phylogenetic information content than mitochondrial genes. On average, nuclear coding sequences contained a higher frequency of Lasso-identified informative sites, and ML phylogenies inferred from nuclear loci showed a higher proportion of branches with strong aLRT support. At the within-species level, the conventional nuclear gene segments also displayed higher frequencies of informative sites and higher average aLRT values, although this same panel performed poorly among congeneric species. Overall, in the absence of whole-genome data, our results support the common practice of combining mitochondrial and nuclear markers in mammalian systematics, both for species delimitation and for resolving phylogenetic relationships among closely related species.

At the level of individual genes, the phylogenetic information content of the widely used mitochondrial genes *CYTB* and *COX1* was comparable to the average of the mitochondrial coding sequences (Supplementary Figure S2). Both genes contain a substantial number of informative sites in their 5′ regions, which supports the common practice of sequencing the 5′ portion of the *COX1* coding region using universal metazoan primers (Folmer et al., 1994). This is further supported by the comparatively higher resolution that both genes provided in phylogenetic inference relative to nuclear coding sequences, as reflected in their generally higher aLRT values (Figure 3b).

Beyond *CYTB* and *COX1*, the *ND5* gene was also effective in resolving phylogenetic relationships at both within- and among-species levels (Supplementary Figure S2), likely due to its longer sequence (1,812 bp) compared with other mitochondrial genes (684–1,542 bp). However, *COX1*, although the second longest sequence analyzed (1,542 bp), provided resolution equivalent or lower than shorter genes such as *CYTB* (1,141 bp) and *ND4* (1,378 bp) in within-species and within-genera datasets, respectively. This supports previous findings that *CYTB* generally provides slightly better resolution than *COX1* for mammalian phylogenies (Tobe et al., 2010). Shorter mitochondrial genes, such as *COX2* and *COX3*, despite containing a relatively high proportion of informative sites, did not achieve high levels of phylogenetic resolution, most likely because of their limited sequence length, which reduces phylogenetic precision. In addition to *CYTB* and *COX1*, the *ND4* and *ND5* genes—although far less commonly employed in mammalian phylogenetics—represent highly suitable markers for addressing phylogenetic questions in mammals. This is consistent with the findings of previous studies that evaluated mammalian deeper divergences (Duchêne et al., 2011) and other metazoan lineages (Zardoya and Meyer, 1996; Creer, 2003; Main et al., 2024).

Traditional Sanger sequencing with overlapping reads produces ∼1,000 bp with high-quality base calling. This has long been regarded as an advantage of sequencing mitochondrial genes, which are generally not much longer than this. For example, the most widely used mitochondrial marker in mammals, *CYTB*, is 1,141 bp in length. In contrast, although the coding sequences of the nuclear genes we analyzed are of similar size (average CDS length = 1,215 bp), their actual genomic lengths are much greater due to the presence of introns. Nevertheless, some of the nuclear genes analyzed warrant particular attention. Specifically, *SNUPN* (1,641 bp), *GPS2* (1,176 bp), and *MBIP* (1,603 bp) exhibited both a high number and a high proportion of phylogenetically informative sites, while the complete gene length was comparable to the mitochondrial genes. To our knowledge, none of these have been employed in multi-gene phylogenetic studies of mammals to date. This highlights the potential of the Lasso regression approach to identify nuclear genes that could serve as valuable markers in future studies aimed at resolving shallow phylogenetic relationships in mammals. A complete list of the analyzed nuclear genes that have CDSs shorter than 1,200 bp and are highly informative is provided in the Supplementary Table S1.

Our analyses did not address potential topological differences among mitochondrial markers, which may result from statistical errors (e.g., limited taxon or site sampling) or model misspecification (e.g., inappropriate substitution models). Nor did they consider topological differences between mitochondrial and nuclear genes (in the case of among-species dataset), which may stem from both biological processes (e.g., incomplete lineage sorting, introgression) and statistical error. Thus, we did not calculate any measure of the success rate of mitochondrial or nuclear genes in recovering taxonomy-based mammalian relationships, because such a statistic can only be formally assessed if the true tree topology is known, which is rarely the case for empirical data. Simulations, however, demonstrate that Lasso regression can efficiently identify sequence sites that significantly contribute to the tree likelihood (Schrago, 2025).

It is also worth noting that mitochondrial genes are less effective for resolving evolutionary divergences at the within-species level. Although the average frequencies of informative sites were similar between the within-species and among-species datasets (∼32%), the average aLRT was much lower for within-species comparisons. This likely reflects differences in the pattern of site informativeness: while both levels yield a comparable number of informative sites, within-species datasets may contain a larger proportion of autapomorphic sites associated with terminal branches, probably due to the presence of transient polymorphisms. Because aLRT and other branch support statistics primarily assess resolution of internal branches, sites containing autapomorphic changes contribute mainly to the tree likelihood (which combines topology and branch lengths) and not to aLRT values. These observations suggest that single mitochondrial genes may be insufficient to resolve population divergences within mammalian species, consistent with previous findings (Morón-López et al., 2022). A suitable strategy may be to combine mitochondrial loci with segments of conventional nuclear markers, which our results show to perform well at the within-species level and to offer complementary information that mitochondrial genes alone cannot capture.

Mitochondrial genes have long been recognized for substantial heterogeneity in substitution rates among codon positions (Brown et al., 1982; Irwin et al., 1991; Yang and Yoder, 2003). Because changes in third codon positions are often synonymous, substitution rates are generally higher than at first and second positions. Our results are consistent with this pattern, as the number of informative sites in third codon positions exceeded that in first and second positions (Supplementary Figure S3). This pattern was particularly pronounced for among-species datasets, where third codon positions contained, on average, 1.8 times more informative sites than first and second positions. The difference in informative sites between first and second positions was minimal, supporting the notion that these positions are under strong purifying selection and justifying their combination into a single partition when analyzing mitochondrial genes. It also demonstrates that third codon positions are not saturated for resolving phylogenetic relationships within mammal genera.

The approach we used to classify alignment sites by informativeness, based on the Lasso regression sparse learning method, is computationally intensive because it requires a very large number of random phylogenetic trees to train the classifier. This limitation prevented us from including a larger number of nuclear genes in the within-genus comparisons. However, because we selected a random sample of nuclear genes that were first filtered based on CDS length—ensuring comparability with mitochondrial CDSs—we believe that this set provides a suitable representation of the overall nuclear CDSs, and that our analyses are unlikely to be biased by this sampling. It is also important to highlight potential future applications of our approach, such as evaluating non-coding genomic regions. This could enable the identification of the most informative genomic regions (e.g., CDSs, introns, regulatory, intergenic) for resolving specific systematic or taxonomic questions, including deeper divergences and other taxonomic clades. Furthermore, as additional genomes become available, it will be possible to compare the performance of mitochondrial and nuclear genomic regions at the within-species level.

Our large-scale evaluation of mammalian mitochondrial and nuclear genes using a sparse learning framework provides the first quantitative benchmark of their relative phylogenetic informativeness. Nuclear loci contained, on average, a higher frequency of informative sites than mitochondrial genes. The overall performances of mitochondrial genes, including the widely sequenced *CYTB* and *COX1*, were comparable to those of nuclear genes. These findings reinforce that, in the absence of whole-genome data, mitochondrial markers are useful for addressing shallow-level taxonomic questions in mammals. Phylogenetic informativeness can be further enhanced by combining mitochondrial genes with conventionally sequenced nuclear segments (e.g., *IRPB*, *BRCA1*), consistent with long-standing practices in mammalian systematics. Moreover, nuclear loci also provide complementary information and are essential when processes such as incomplete lineage sorting, recombination, or hybridization are relevant.

By integrating methodological innovation with extensive comparative datasets, our study provides a principled foundation for marker selection in mammalian taxonomy, species delimitation, and evolutionary research, while offering a broadly applicable framework that can be deployed in any lineage. Together, these contributions pointing toward a more data-driven framework for systematics in the genomic era. Users interested in applying the workflow to their own datasets may download the Python implementation at http://github.com/cschrago/LassoTrim.

## Data Availability

The original contributions presented in the study are included in the article/Supplementary Material, further inquiries can be directed to the corresponding authors.
