# Supplementary material for: Assessment of phylogenetic informativeness in mitochondrial and nuclear genes for mammalian systematics using sparse learning
Source: Front Bioinform. 2026 Jan 8;5:1704212. doi: 10.3389/fbinf.2025.1704212 (PMC12824000; doi:10.3389/fbinf.2025.1704212)
Supplement: Supplementary file 1 [file DataSheet1.docx]

***Supplementary Material of***

**Assessment of Phylogenetic Informativeness in Mitochondrial and Nuclear Genes for Mammalian Systematics Using Sparse Learning**

Carlos G. Schrago and Beatriz Mello

**Figure S1**: Comparison of the mean information content inferred by Lasso for mitochondrial and nuclear genes using the within-genera datasets. (a) Distribution of the percentage of informative sites inferred for mitochondrial and nuclear genes. (b) Mean difference in the percentage of informative sites between mitochondrial and nuclear genes. Dashed lines indicate the 95% confidence interval for the difference, which includes zero, indicating no detectable difference between the two groups.

**Figure S2**: Frequency of alignment-informative sites in mitochondrial genes inferred by Lasso regression (top panels) for within-species (top-left) and among-species (top-left). Each point represents an alignment, showing the proportion/number of sites inferred as informative by Lasso regression. Average aLRT values (bottom panels) for within-species (bottom-left) and among-species (bottom-right) datasets for the analyzed mitochondrial genes. Each point represents an alignment, showing the mean of the aLRT values estimated in IQ-TREE for that alignment.

**Figure S3:** Distribution of informative sites by codon position—first (blue), second (green), and third (red)—for within-species (a) and among-genera (b) datasets.

**Table S1**: Top-informative nuclear genes. Complete list of the analyzed nuclear genes that have CDSs shorter than 1,200 bp and are highly informative.

| Top-informative nuclear genes | Ensembl transcript accesion |
| --- | --- |
| FKBP3 | ENST00000216330 |
| ABHD11 | ENST00000222800 |
| EMC3 | ENST00000245046 |
| SHFL | ENST00000253110 |
| MNAT1 | ENST00000261245 |
| SEC22C | ENST00000264454 |
| EXOSC7 | ENST00000265564 |
| SETD7 | ENST00000274031 |
| GPM6A | ENST00000280187 |
| LRRC2 | ENST00000296144 |
| KLF7 | ENST00000309446 |
| BVES | ENST00000314641 |
| HCCS | ENST00000321143 |
| YBX1 | ENST00000321358 |
| CENPA | ENST00000335756 |
| FIBP | ENST00000338369 |
| RAD1 | ENST00000341754 |
| NTPCR | ENST00000366627 |
| LPGAT1 | ENST00000366997 |
| NEK7 | ENST00000367385 |
| GCLM | ENST00000370238 |
| SDHB | ENST00000375499 |
| NUDT5 | ENST00000378937 |
| APTX | ENST00000379817 |
| GPS2 | ENST00000380728 |
| MBIP | ENST00000416007 |
| TRAPPC4 | ENST00000533058 |
| CYB5RL | ENST00000534324 |
| GTF2H3 | ENST00000543341 |
| SNUPN | ENST00000567134 |
| MRPL28 | ENST00000648346 |
| GKN1 | ENST00000673932 |
